# Synthesis, Spectroscopy, Thermal Analysis, Magnetic Properties and Biological Activity Studies of Cu(II) and Co(II) Complexes with Schiff Base Dye Ligands

**DOI:** 10.3390/molecules17066434

**Published:** 2012-05-29

**Authors:** Raziyeh Arab Ahmadi, Saeid Amani

**Affiliations:** Chemistry Department, Faculty of Sciences, Arak University, Dr. Beheshti Ave., Arak 38156-88349, Iran; Email: r-arabahmadi@phd.araku.ac.ir

**Keywords:** Cu(II) and Co(II) complexes, spectroscopic characterization, magnetic moment studies, antibacterial studies

## Abstract

Three azo group-containing Schiff base ligands, namely 1-{3-[(3-hydroxy-propylimino)methyl]-4-hydroxyphenylazo}-4-nitrobenzene (**2a**), 1-{3-[(3-hydroxypropyl-imino)methyl]-4-hydroxyphenylazo}-2-chloro-4-nitrobenzene (**2b**) and 1-{3-[(3-hydroxy-propylimino)methyl]-4-hydroxyphenylazo}-4-chloro-3-nitrobenzene (**2c**) were prepared. The ligands were characterized by elemental analysis, FTIR spectroscopy, UV-Vis spectroscopy, ^13^C- and ^1^H-NMR spectroscopy and thermogravimetric analysis. Next the corresponding copper(II) and cobalt(II) metal complexes were synthesized and characterized by the physicochemical and spectroscopic methods of elemental analysis, FTIR spectroscopy, UV-Vis spectroscopy, magnetic moment measurements, and thermogravimetric analysis (TGA) and (DSC). The room temperature effective magnetic moments of complexes are 1.45, 1.56, 1.62, 2.16, 2.26 and 2.80 B.M. for complexes **3a**, **3b**, **3c**, **4a** **4b**, and **4c**, respectively, indicating that the complexes are paramagnetic with considerable electronic communication between the two metal centers.

## 1. Introduction

Azo Schiff base complexes contain both azo and azomethine groups. The azo group possesses excellent donor properties and is important in coordination chemistry [1,2,3], and some azo compounds have been shown to possess good antibacterial activity [4,5]. Schiff bases are well known to have antifungal, antitumor and herbicidal activities [6,7,8,9,10,11]. Salicylaldimine-based ligands have found applications in preparation of metallomesogens, optical metal ion detection and enantioselective catalysis [12,13,14,15]. Azo Schiff bases are commonly synthesized by coupling a diazonium reagent with an aromatic aldehyde to form an azo aldehyde [16,17]. The azomethine group has good donor properties and can form stable complexes with transition metal ions [18,19,20]. The azo and azomethine groups on azo Schiff base ligands are oriented in such a way that coordination of both groups to a metal ion is not possible, thus, preferential coordination of the azomethine group while the azo group is left free and uncoordinated has been observed [21,22,23,24]. Schiff bases derived from salicylaldehydes are known polydentate ligands, coordinating to metals in both their deprotonated and neutral forms [25,26]. Some cobalt and copper complexes exhibit diverse biological properties viz. anti-inflammatory, antibacterial and anticancer, *etc.* [27].

Many Schiff base complexes show excellent catalytic activity for various reactions at high temperatures (>100 °C) and in the presence of water. Over the past few years, there have been many reports on their applications in homogeneous and heterogeneous catalysis [28,29]. A wide variety of cobalt(II) and copper(II) complexes are known to bind dioxygen more or less reversibly and are therefore are frequently studied as model compounds for natural oxygen carriers and for O_2_ storage [30]. Some thermochemical work on a limited series of cobalt(II) and copper(II) complexes has been reported by various researchers [31,32]. Based on the aforementioned properties of Schiff bases and azo compounds, we reported herein the synthesis and spectroscopic studies as well as thermal investigation of novel salicylaldimine-based ligands 1-{3-[(3-hydroxypropyl-imino)methyl]-4-hydroxyphenylazo}-4-nitrobenzene (**2a**), 1-{3-[(3-hydroxypropyl-imino)methyl]-4-hydroxyphenyl-azo}-2-chloro-4-nitrobenzene (**2b**) and 1-{3-[(3-hydroxypropyl-imino)methyl]-4-hydroxyphenylazo}-4-chloro-3-nitrobenzene (**2c**). ^13^C- and ^1^H-NMR spectra were obtained to determine the structure of the ligands **2a**–**c**. The copper(II) and cobalt(II) complexes derived from azo-linked salicylaldimine were also prepared and their structures were confirmed by elemental analysis, FTIR spectroscopy, UV-Vis spectroscopy, thermogravimetric analysis and magnetic moment measurements.

## 2. Results and Discussion

### 2.1. Biological Studies

Three ligands and six complexes were screened for their activity against *Escherichia coli* (Gram negative), *Staphylococcus aureus* (Gram positive), *Micrococcus luteus*, *Enterobacter cloacae*, *Klebsiella pneumoniae* and antifungal activities against *Aspergillus niger* and *Pencillium*. The inhibition zones were measured and compared with standard drugs. The antibacterial and antifungal activities of the new compounds are presented in Table 1. The results indicate that the ligands have no activity against fungi and have some activity against the bacteria. The complexes have no activity against fungi, except for complex **3a**. complex **3b** has no activity against bacteria, while complexes **3a,c** have activity against bacteria. The complexes **4a**–**c** have activity against bacteria.

**Table 1:** Antimicrobial activity studies of ligands and their metal complexes.

Compound	*E. coli*	*S. aureus*	*M. luteu*	*E. cloacae*	*K. pneumoniae*	*A. niger*	*Pencillium*
**2a**	4	3	0	0	0	0	0
**2b**	3	4	0	0	0	0	0
**2c**	5	3	3	3	2	0	0
**3a**	0	6	3	0	0	0	4
**3b**	0	0	0	0	0	0	0
**3c**	0	2	0	0	0	0	0
**4a**	5	2	1	0	0	0	0
**4b**	0	0	0	0	2	0	0
**4c**	0	0	2	0	0	0	0

### 2.2. Infrared Spectra

The infrared spectra of the free ligands and the metal complexes were obtained over a spectral range of 4,000–300 cm^−1^. A comparison of the infrared spectra of the ligands and the respective complexes reveals the absence of absorption bands associated with the O-H stretching of the phenol groups, indicating the loss of the protons upon complexation, along with formation of a metal-oxygen bond. This finding was further supported by an increase in (C-O) frequency in the spectra of the metal complexes compared to the ligands [33]. The bands at 1,647–1,651 cm^−1^ were assigned to the stretching vibration of the azomethine group of the ligands **2a**–**c**. This band is shifted in the complexes toward lower frequencies. Reduction of the double bond character of the C=N bond, which is caused by the coordination of nitrogen into the metal center, is in agreement with results obtained from similar complexes [13,16,34]. Based on data from earlier reports, we could assign the bands at 408–443 and 524–599 cm^−1^ to M-N and M-O vibrations, respectively [35].

### 2.3. Electronic Spectra

The features of the electronic spectra of the cobalt(II) complexes are very similar to each other, and they are typical for tetrahedral high-spin cobalt(II) complexes. The electronic structures of cobalt(II) complexes with different ligands have been presented in the literature [36,37]. Based on the simplest model, three spin-allowed crystal field bands are expected in the spectra of tetrahedral cobalt(II) complexes, *i.e*.,^4^A_2_(F) → ^4^T_2_(F), ^4^A_2_(F) → ^4^T_1_(F), ^4^A_2_(F) → ^4^T_1_(P). Usually this type of cobalt(II) complex exhibits two bands between 610 nm and 830 nm that can be assigned to the ^4^A_2_(F) → ^4^T_2_(F) and ^4^A_2_(F) → ^4^T_1_(F) transitions, respectively. The transition ^4^A_2_(F) → ^4^T_1_(P) is usually observed as a well-defined shoulder at approximately 550 nm [38]. The electronic spectra of the complexes were obtained from solid samples using the diffuse reflectance technique. The cobalt(II) complexes each show a broad shoulder with three bands (605, 489 and 278 nm for **4a**, 622, 508 and 270 nm for **4b**, and 528, 438 and 285 nm for **4c**). These bands are assigned as spin-allowed crystal field transitions. Absorption bands are observed at 278, 270 and 285 nm for in the spectra of **4a**, **4b** and **4c**, respectively. These bands are attributed to charge transfer from the non-bonding orbitals of the oxygen atoms in the ligand to the cobalt(II) d orbitals. The last absorption band at approximately 262 nm is assigned to the π → π* or n → π* transitions of the ligand [39,40,41]. The spectra of the copper(II) complexes display broad bands at 457 nm for complex **3a**, 610 nm for complex **3b** and 547 nm for complex **3c** due to the ligand field transition for the CuNO_3_ chromophore [42,43]. The second absorption bands at 360, 384 and 441 nm for compounds **3a**, **3b** and **3c**, respectively, are assigned to charge transfer from the non-bonding orbital of the oxygen atoms to the vacant copper(II) d orbitals [42,44]. The last absorption band observed at approximately 265 nm for each of the complexes is associated with π → π* or n → π* transitions of the ligand [45].

### 2.4. Magnetic Moment

The magnetic moments of the cobalt(II) and copper(II) complexes were determined by the Evans method [46]. This method is based on the principle that the position of a given proton resonance (*i.e*., t-butyl alcohol) in the NMR spectrum of a molecule is dependent on the bulk volume magnetic susceptibility of the medium in which the molecule is found. The shift of the proton resonance for an inert substance due to the presence of paramagnetic ions is given by theoretical expression (1):



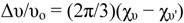
(1)


In this equation, Δυ is the shift, υ_o_ is the applied field, χ_υ_ is the volume magnetic susceptibility of the solution containing paramagnetic ions and χ_υ'_ is the volume magnetic susceptibility of the reference solution. The effective magnetic moments (μ_eff_) of the compounds in this study were determined at room temperature to be 1.45, 1.56, 1.62, 2.16, 2.26 and 2.80 B.M. for **3a**, **3b**, **3c**, **4a**, **4b** and **4c**, respectively. These values were calculated using the spin only equation, μ_eff_ = 2.82[T χ^corr^ − Nα]^1/2^, where T is the absolute temperature, χ^corr^ is the molar magnetic susceptibility corrected for paramagnetism of the constituent atoms, and Nα is the temperature independent pararmagnetism. The magnetic moment of the cobalt(II) complexes at room temperature were found to be 2.16 to 2.80 B.M. range per cobalt(II) ion, which appears to be low for a d^7^ high spin configuration, indicating that there must be a strong spin-spin interaction through the bridging ligands [47]. The values of μ_eff_ for the copper(II) complexes at room temperature were found to be in the range of 1.45–1.64 B.M. per copper(II) ion. These data are low for a d^9^ configuration. The subnormal magnetic moment was ascribed to a significant electronic coupling between the two copper(II) centers, postulated to occur through the bridging atoms (through a superexchange pathway) [48,49].

### 2.5. ^13^C- and ^1^H-NMR Spectra of the Ligands

^1^H- and ^13^C-NMR spectra of the ligands were recorded in DMSO-d_6_. In the ^1^H-NMR spectra of the ligands, the singlet resonances in the 14–13.21 and 4.7–4.79 ppm regions can be attributed to the protons of the phenolic and alcoholic OH groups, respectively. All three ligands exhibit singlet signals in the range of 8.2–8.76 ppm, which were attributed to the azomethine group protons. In addition, the multiple signals in the range of approximately 3.7–1.83 ppm were attributed to aliphatic protons. The signals of ^13^C-NMR of the ligands **2a**, **2b** and **2c**, were appeared in the range of 32.8–58.2 and 115.3–166.7 ppm are assigned to aliphatic and aromatic carbon, respectively. The signal in the range of 176–178.7 ppm, can be assigned to C=N carbon. All ^1^H- and ^13^C-NMR of the ligands are attached as the supplementary materials.

### 2.6. Thermal Analysis

**Figure 1:**
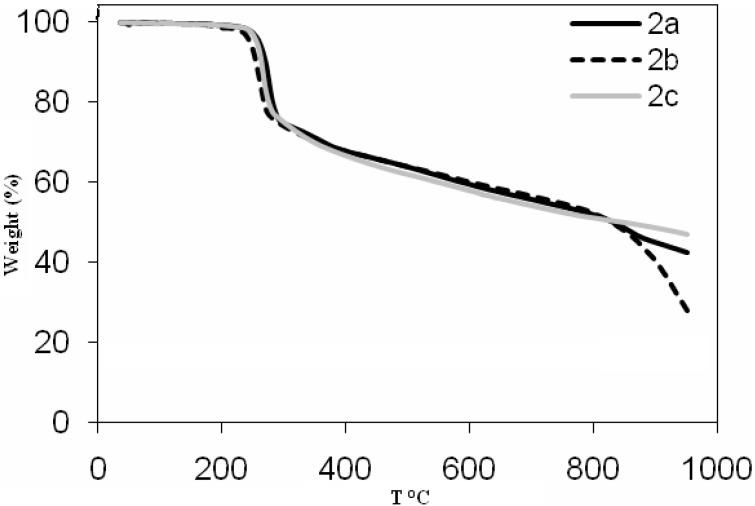
Thermogravimetric curves of ligands **2a**–**c**.

**Figure 2:**
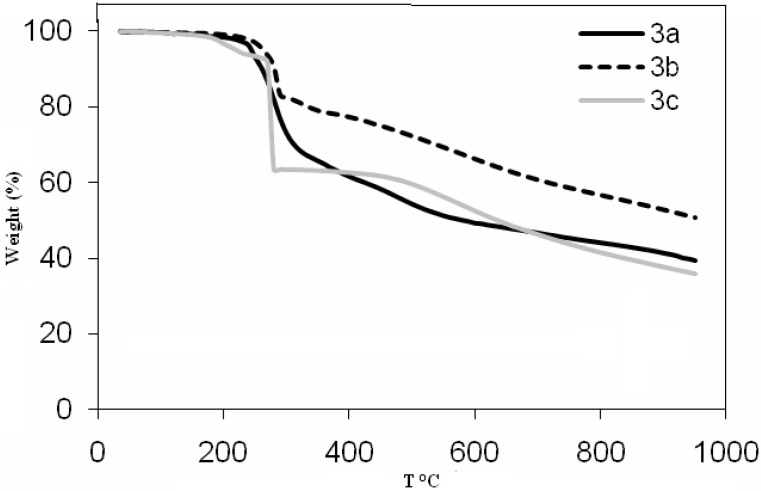
Thermogravimetric curves for copper(II) complexes **3a**–**c**.

The TGA curves (Figure 1 and Figure 2) indicate that the ligands **2a**, **2b** and **2c** begin to decompose at 277, 260 and 268 °C, respectively. Comparison of the decomposition temperature of the ligands shows that **2a** and **2c** decompose at higher temperatures than **2b**. The TGA curve for **2a** displays three stages of mass loss within the temperature range of 220–925 °C. The first stage is at 220–320 °C, and exhibits a mass loss of 26.13%, corresponding to the loss of C_4_H_6_N_2_O (calc. 26.92%). The second stage occurs at 320–790 °C, with a mass loss of 21.11%, corresponding to the loss of C_6_H_4_ (calc. 20.08%). The third stage of decomposition occurs at the temperature range 790–925 °C, with a mass loss of 8.7%, corresponding to the loss of NO (calc. 8.2%).

Complex **2b** shows similar degradation behavior and displays three stages of mass loss within the temperature range of 230–953 °C. The first stage is at 230–300 °C, with a mass loss of 24.28%, corresponding to the loss of C_5_H_10_N_2_O (calc. 25.19%). The second stage occurs at 300–780 °C, with a mass loss of 21.07%, corresponding to the loss of C_6_H_4_O (calc. 20.33%). The third stage of decomposition occurs at the temperature range 780–953 °C, with a mass loss of 27.32%, corresponding to the loss of C_6_H_3_NO_2_ (calc. 26.74%). The TG curve for **2c** exhibits to two stages of mass loss within the temperature range of 243–953 °C. The first stage occurs at 243–329 °C, with a mass loss of 26.09%, corresponding to the loss of C_5_H_8_NO (calc. 27.03%). The second stage of decomposition occurs at 329–953 °C and is roughly assigned to the loss of C_6_H_5_O with a mass loss 25.43% (calc. 25.66%). The TG curves indicate that the copper complexes **3a**, **3b** and **3c** begin decomposition at 283, 299 and 308 °C, respectively. A comparison of the thermogravimetric curves for the ligands and the copper complexes shows that the copper complexes are more thermally stable than the azo dye ligands. The order of thermal stability is found to be **3c** > **3b** > **3a** > **2a** > **2c** > **2b**. The organic portion of the copper complexes may decompose in three or four steps. The decompositions of the copper complexes all ended with formation of CuO [50,51]. The thermogravimetric analysis data for the ligands and the copper(II) complexes are presented in Table 2. Metal complexes derived from salicylideneamine are among the best known classes of metallomesogens. The liquid crystalline behavior of all the cobalt(II) complexes was studied by thermal analysis (DSC). The phase transitions and thermodynamic data for the complexes were summarized in Table 3. Surprisingly, none of the cobalt(II) complexes displayed any mesomorphic properties. The lack of liquid crystallinity is generally believed to be attributed to weaker interaction between the cobalt complexes.

**Table 2:** Thermogravimetric analysis data for the ligands and copper(II) complexes.

Compound	Temperature range (°C)	TG weight loss % calc./found	Assignments
**2a**	220–320	26.92 (26.13)	C_4_H_6_N_2_O
	320–790	20.08 (21.11)	C_6_H_4_
	790–925	8.2 (8.7)	NO
**2b**	230–300	25.19 (24.28)	C_5_H_10_N_2_O
	300–780	20.33 (21.07)	C_6_H_4_O
	780–953	26.74 (27.32)	C_6_H_3_NO_2_
**2c**	243–329	27.03 (26.09)	C_5_H_8_NO
	329–953	25.66 (25.43)	C_6_H_5_O
**3a**	230–335	31.96 (31.04)	C_12_H_8_N_4_O_4_
	335–650	19.51 (19.33)	C_11_H_6_N_2_
	650–951	9.3 (9.1)	CuO
**3b**	210–295	12.14 (11.22)	C_6_H_3_N_2_
	295–710	23.17 (22.78)	C_9_H_7_NO_2_Cl
	710–953	9.3 (9.7)	CuO
**3c**	165–230	5.15 (4.59)	C_3_H_7_N
	230–310	30.86 (31.16)	C_12_H_6_N_4_O_4_Cl_2_
	310–750	20.27 (19.95)	C_12_H_6_N_3_O_2_
	750–953	7.19 (8.07)	CuO

**Table 3:** Transition temperatures and enthalpy changes for the cobalt(II) complexes.

Compound	Transition	T/°C	ΔH/J g^−1^
**4a**	Cr-I (dec)	293	−147
	Cr_1_-Cr_2_	168	1.61
**4b**	Cr_2_-Cr_3_	189	5.27
	Cr_3_-I (dec)	248	−141
**4c**	Cr_1_-Cr_2_	191	1.2
	Cr_2_-I (dec)	230	−3.54

Cr = crystal, I = Isotropic liquid, dec = decomposition.

## 3. Experimental

### 3.1. Physical Measurements

C, H and N composition determinations were undertaken using an Elemental Analysis System GmbH Vario EL II. Cobalt and copper determinations were performed using a Perkin-Elmer 2380 Atomic Absorption Spectrophotometer. Electronic spectra of complexes were recorded on a Perkin-Elmer Lambda 900 spectrophotometer using the diffuse reflectance technique; MgO was used as a reference. Electronic spectra of ligands in DMSO were recorded on a Perkin-Elmer Lambda 15 instrument. FTIR spectra of the compounds as KBr disks were obtained in the 4,000–300 cm^−1^ range with a Galaxy series FTIR 5000 spectrophotometer. The spectra were calibrated using the polystyrene bands at 3,028, 1,601 and 1,208 cm^−1^. The room temperature magnetic moment of each metal complex was measured according to the Evans method [46]. ^13^C- and ^1^H-NMR spectroscopy was recorded using a Bruker 300 MHz spectrometer. Thermal analyses were performed using a Perkin-Elmer Thermogravimetric Analyzer TG/DTA 6300 under a N_2_ gas flow (20 mL min^−1^) at ambient pressure. A heating rate of 10 °C min^−1^ was chosen. In the cases where the TG curve indicated the possibility of stable intermediates, a heating rate of 5 °C min^−1^ or 1 °C min^−1^ was applied.

### 3.2. Materials

Chemicals: All chemicals and solvents were of reagent grade quality and were purchased from Merck Chemical Company and used as received without further purification, except for vaccum dring over P_2_O_5_.

### 3.3. Synthesis of the Starting Materials

*1-(3-Formyl-4-hydroxyphenylazo)-4-nitrobenzene* (**1a**). Azo dye **1a** was synthesized according to the well-known published procedure [52]. A suspension of 4-nitroaniline (5.52 g, 40 mmol) in hydrochloric acid (36 mL) and water (16 mL) was heated to 70 °C until complete dissolution. The clear solution was poured into ice water and was diazotized below 5 °C with sodium nitrite (2.8 g, 40 mmol) dissolved in water (10 mL). The cold diazonium solution was added over the course of 30 min at 0 °C to a solution of salicylaldehyde (4.26 mL, 40 mmol) in water (75 mL) containing sodium hydroxide (1.6 g) and sodium carbonate (14.8 g). During the addition process, the solution was vigorously stirred. The product was collected by vacuum filtration and washed with NaCl solution (100 mL, 10%). Coupling of the diazonium reagent to the salicylaldehyde occurred at the position *para* to the hydroxyl group. The diazo compound was recrystallized several times from ethyl alcohol. C_13_H_9_N_3_O_4_, yellow solid, yield: 90%, m.p.: 185–186 °C. FTIR (KBr, cm^−1^): 3,101 (-OH group), 1,664 (-CHO group), 1,479 (N=N), 1,346 (NO_2_ group), 1,286 (C-O) cm^−1^. UV-Vis: λ_max_ = 397, 547 nm. ^1^H-NMR (DMSO): δ = 11.49 (1H, s), 10.08 (1H, s), 8.4 (2H, t, *J* = 7.14 Hz), 8.3 (1H, d, *J* = 2.34 Hz), 8.22 (1H, q, *J* = 2.37 Hz), 8.01 (2H, t, *J* = 7.08 Hz), 7.1 (1H, d, *J* = 9 Hz) ppm.

*1-(3-Formyl-4-hydroxyphenylazo)-2-chloro-4-nitrobenzene* (**1b**). Compound **1b** was synthesized from 2-chloro-4-nitroaniline (7.9 g, 45 mmol) following the procedure given above for **1a**, using the same molar ratio of the reagents. The purity of the compound was evaluated using thin layer chromatography. C_13_H_8_N_3_O_4_Cl, yellow solid, yield: 85%, m.p.: 127–128 °C. FTIR (KBr, cm^−1^): 3,431 (-OH group), 1,660 (-CHO group), 1,481 (N=N), 1,346 (NO_2_ group), 1,286 (C-O) cm^−1^. UV-Vis: λ_max_ = 380, 566 nm. ^1^H-NMR (DMSO): δ = 10.35 (1H, s), 8.5 (1H, s), 7.1 (1H, d, *J* = 9.28 Hz), 7.8 (1H, d, *J* = 8.78 Hz), 8.1 (1H, d, *J* = 8.76 Hz), 8.23 (1H, s), 8.3 (2H, d, *J* = 8.9 Hz) ppm.

*1-(3-Formyl-4-hydroxyphenylazo)-4-chloro-3-nitrobenzene* (**1c**). Compound **1c** was synthesized from 4-chloro-3-nitroaniline (7.9 g or 45 mmol) following the procedure given above for **1a**, using the same molar ratio of the reagents. The purity of the compound was evaluated using thin layer chromatography. C_13_H_8_N_3_O_4_Cl, yellow solid, yield: 78%, m.p.: 243–244 °C. FTIR (KBr, cm^−1^): 3,428 (-OH group), 1,658 (-CHO group), 1,473 (N=N), 1,550 (NO_2_ group), 1,284 (C-O) cm^−1^. UV-Vis: λ_max_ = 355, 490 nm. ^1^H-NMR (DMSO): δ = 11.8 (1H, s), 10.36 (1H, s), 8.4 (1H, s), 8.21 (1H, s), 8.1 (2H, d, *J* = 9.5 Hz), 7.9 (1H, d, *J* = 8.3 Hz), 7.2 (1H, d, *J* = 8.4 Hz) ppm.

### 3.4. Synthesis of the Schiff Base Ligands

Compounds **2a**–**c** were prepared using a method previously reported in the literature [53]. For each ligand, a mixture of 0.02 mol of 3-amino-1-propanol and 0.02 mol of the corresponding azo dye **1a**–**c** was dissolved in absolute ethanol (80 mL) with a few drops of glacial acetic acid as a catalyst. The resulting mixture was allowed to stir under reflux for 2–3 h. The product was vacuum-filtered and washed with a small amount of hot ethanol. The products were soluble in solvents such as DMF and DMSO.

*1-{3-[(3-Hydroxypropylimino)methyl]-4-hydroxyphenylazo}-4-nitrobenzene* (**2a**). Yellow solid, yield: 80%, m.p.: 168–170 °C. Anal. Calc. for C_16_H_16_N_4_O_4_. 2H_2_O, C: 52.7, H: 5.5, N: 15.3. Found: C: 52.8, H: 6.4, N: 14.8%. FTIR (KBr, cm^−1^): 3,100–3,252 (-OH group), 3,051 (C-H, aromatic), 2,922, 2,841 (C-H, aliphatic), 1,647 (C=N), 1,608 (C=C, aromatic), 1,421 (N=N), 1,273 (C-O, phenolic) cm^−1^. UV-Vis: λ_max_ = 269 (9,296), 399 (12,933), 462 (10,766) nm. ^1^H-NMR (DMSO): δ = 13.21 (1H, s), 8.72 (1H, s), 8.36 (2H, s), 8.09 (1H, s), 7.92 (3H, s), 6.67 (1H, s), 4.73 (1H, s), 3.72(1H), 3.5 (1H), 1.83 (1H) ppm. ^13^C-{^1^H}NMR (DMSO) δ = 32.9, 50.5, 58.3, 115.6, 123, 123.5, 125.4, 126.6, 136.4, 141.8, 147.6, 156.3, 166.9, 176.5 ppm.

*1-{3-[(3-Hydroxypropylimino)methyl]-4-hydroxyphenylazo}-2-chloro-4-nitrobenzene* (**2b**). Light brown solid, yield: 71%, m.p.: 187–190 °C. Anal. Calc. for C_16_H_15_N_4_O_4_Cl. 5H_2_O, C: 42.4, H: 5.4, N: 12.3. Found: C: 42, H: 5.2, N: 11.83%. FTIR (KBr, cm^−1^): 3,100–3,271 (-OH group), 3,057 (C-H, aromatic), 2,958, 2,897 (C-H, aliphatic), 1,649 (C=N), 1,610 (C=C, aromatic), 1,450 (N=N), 1,271 (C-O, phenolic) cm^−1^. UV-Vis: λ_max_ = 268 (825), 412 (12,030), 530 (16,645) nm. ^1^H-NMR (DMSO): δ = 13.83 (1H, s); 8.76 (1H, s); 8.4 (1H, s), 8.26 (1H, s), 8.12 (1H, s), 7.94 (1H, s), 7.78 (1H, s), 6.67 (1H, s), 4.79 (1H, s), 3.7 (1H), 3.5 (1H), 1.83 (1H) ppm. ^13^C-{^1^H}NMR (DMSO) δ = 32.8, 49.8, 58.2, 115.3, 118.6, 123.8, 124.5, 126.1, 126.7, 132.8, 138.8, 141.9, 147.4, 152.6, 167.2, 178.7 ppm.

*1-{3-[(3-Hydroxypropylimino)methyl]-4-hydroxyphenylazo}-4-chloro-3-nitrobenzene* (**2c**). Light brown solid, yield: 46%, m.p.: 121–125 °C. Anal. Calc. for C_16_H_15_N_4_O_4_Cl. C: 52.9, H: 4.1, N: 15.4. Found: C: 52.4, H: 4.4, N: 15.1%. FTIR (KBr, cm^−1^): 3,300 (-OH group), 3,090 (C-H, aromatic), 2,922, 2,877 (C-H, aliphatic), 1,651 (C=N), 1,612 (C=C, aromatic), 1,462 (N=N), 1,275 (C-O, phenolic) cm^−1^. UV-Vis: λ_max_ = 264 (15,463), 417 (19,260), 465 (18,703) nm. ^1^H-NMR (DMSO): δ = 14 (1H, s), 8.3 (1H, s), 8.1 (1H, d), 8 (2H, t), 7.9 (2H, d), 6.71 (1H, d), 4.7 (1H, s), 3.7 (1H), 3.5 (1H), 1.8 (1H) ppm. ^13^C-{^1^H}NMR (DMSO) δ = 33, 50.7, 58.3, 115.7, 118, 123.2, 125.5, 126.5, 127.4, 133, 135.8, 141.4, 148.6, 151.6, 166.7, 176 ppm.

### 3.5. Synthesis of the Copper(II) Complexes

The copper complexes were prepared following a previously published procedure [54]. For each complex, an alcoholic solution containing 10 mmol of 3-amino-1-propanol was added to a solution of the corresponding azo dye **1a–c** (10 mmol) in boiling methanol (50 mL). Then, sodium acetate (10 mmol) dissolved in water (10 mL) was added to the reaction. Finally, boiling methanol solution (50 mL) containing Cu(CH_3_COO)_2_•H_2_O (10 mmol) was added. The stirring mixture was refluxed for 2 h and then the product was vacuum-filtered and washed with a small amount of ethanol.

Complex *[(1-{3-[(3-Hydroxypropylimino)methyl]-4-hydroxyphenylazo}-4-nitrobenzenato)_2_(H_2_O)_4_Cu_2_]* (**3a**). Dark orange solid, yield: 73%, Anal. Calc. for C_32_H_28_N_8_O_8_Cu_2_. 4 H_2_O, C: 45.1, H: 4.2, N: 13.15, Cu: 14.91. Found: C: 45.6, H: 4.3, N: 13.13, Cu: 14.7%. FTIR (KBr, cm^−1^): 1,626 (C=N), 1,473 (N=N), 1,334 (C-O, phenolic), 422 (Cu-N), 599 (Cu-O) cm^−1^. UV-Vis: λ_max_ = 278, 360 and 457 nm.

Complex *[(1-{3-[(3-Hydroxypropylimino)methyl]-4-hydroxyphenylazo}-2-chloro-4-nitrobenzenato)_2_Cu_2_]* (**3b**). Brownish red solid, yield: 65%, Anal. Calc. for C_32_H_26_N_8_O_8_Cu_2_Cl_2_, C: 45.3, H: 3.0, N: 13.2, Cu: 14.97. Found: C: 45.16, H: 1.991, N: 12.52, Cu: 15.4%. FTIR (KBr, cm^−1^): 1,624 (C=N), 1,475 (N=N), 1,334 (C-O, phenolic), 426 (Cu-N), 563 (Cu-O) cm^−1^. UV-Vis: λ_max_ = 271, 472 and 620 nm.

Complex *[(1-{3-[(3-Hydroxypropylimino)methyl]-4-hydroxyphenylazo}-4-chloro-3-nitrobenzenato)_2_(CHCl_3_)_2_(H_2_O)_2_Cu_2_]* (**3c**). Light brown solid, yield: 42%, Anal. Calc. for C_32_H_26_N_8_O_8_Cu_2_Cl_2_. 2CHCl_3_•H_2_O, C: 36.9, H: 2.7, N: 10.1, Cu: 11.48. Found: C: 36.4, H: 2.4, N: 10.02, Cu: 12.4%. FTIR (KBr, cm^−1^): 1,620 (C=N), 1,471 (N=N), 1,336 (C-O, phenolic), 443 (Cu-N), 524 (Cu-O) cm^−1^. UV-Vis: λ_max_ = 265, 441 and 546 nm.

### 3.6. Synthesis of the Cobalt(II) Complexes

The cobalt complexes were prepared according to the procedure described above for the copper complexes using Co(CH_3_COO)_2_•H_2_O instead of Cu(CH_3_COO)_2_•H_2_O.

Complex *[(1-{3-[(3-Hydroxypropylimino)methyl]-4-hydroxyphenylazo}-4-nitrobenzenato)_2_(C_2_H_5_OH)_2_Co_2_]* (**4a**). Brownish red solid, yield: 49%, Anal. Calc. for C_32_H_28_N_8_O_8_Co_2_. 2CH_3_CH_2_OH, C: 50.13, H: 4.63, N: 12.98, Co: 13.68. Found: C: 50.30, H: 5.83, N: 13.87, Co: 14.34%. FTIR (KBr, cm^−1^): 1,635 (C=N), 1,479 (N=N), 1,336 (C-O, phenolic), 408 (Co-N), 576 (Co-O) cm^−1^. UV-Vis: λ_max_ = 256, 278, 489 and 605 nm.

Complex *[(1-{3-[(3-Hydroxypropylimino)methyl]-4-hydroxyphenylazo}-2-chloro-4-nitrobenzenato)_2_(C_2_H_5_OH)Co_2_]* (**4b**). Brown solid, yield: 65%, Anal. Calc. for C_32_H_26_N_8_O_8_Co_2_Cl_2_. CH_3_CH_2_OH, C: 46.12, H: 3.61, N: 12.65 Co: 13.32. Found: C: 46.93, H: 3.298, N: 13.17, Co: 12.72%. FTIR (KBr, cm^−1^): 1,641 (C=N), 1,479 (N=N), 1,344 (C-O, phenolic), 414 (Co-N), 584 (Co-O) cm^−1^. UV-Vis: λ_max_ = 257, 270, 508 and 622 nm.

Complex *[(1-{3-[(3-Hydroxypropylimino)methyl]-4-hydroxyphenylazo}-4-chloro-3-nitrobenzenato)_2_(CHCl_3_)_2_(H_2_O)_2_Cu_2_]* (**4c**). Yellow solid, yield: 34%, Anal. Calc. for C_32_H_26_N_8_O_8_Co_2_Cl_2_. 2CHCl_3_•H_2_O, C: 37.24, H: 2.73, N: 10.21, Co: 10.76. Found: C: 37.3, H: 3.071, N: 10.14, Co: 11.2%. FTIR (KBr, cm^−1^): 1,602 (C=N), 1,475 (N=N), 1,330 (C-O, phenolic), 443 (Co-N), 592 (Co-O) cm^−1^. UV-Vis: λ_max_ = 259, 285, 438 and 528 nm.

**Scheme 1:**
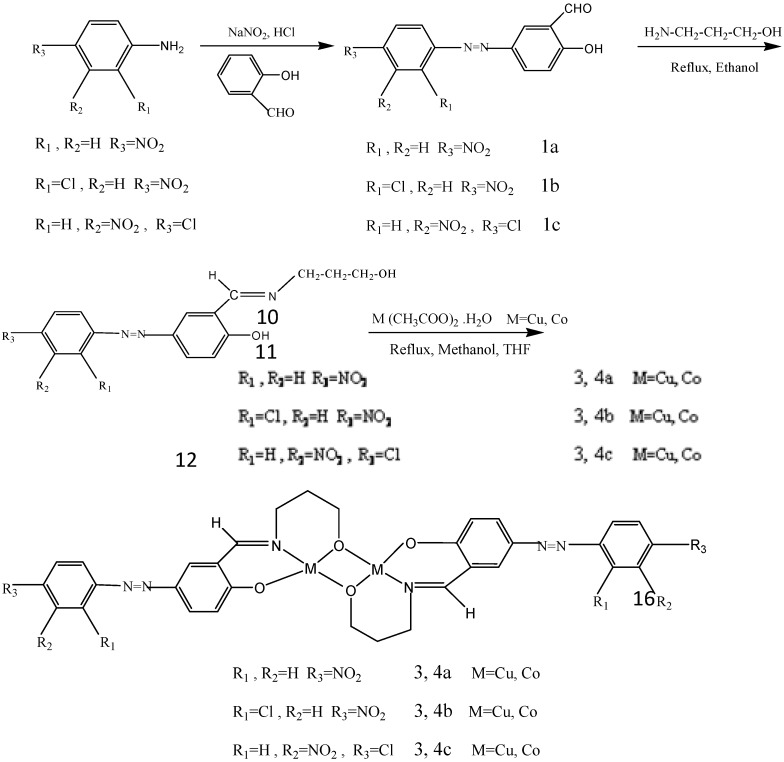
Preparation of ligands and complexes.

The various synthetic reactions are summarized below (Scheme 1).

### 3.7. Biological Studies

The antimicrobial activities of the compounds in this study were tested by the disc diffusion method [55]. *Mueller Hinton Agar* (MHA, Oxoid, 15 mL) and *Sabouraud Dextrose Agar* (SDA, sterilized in a flask and cooled to 45–50 °C) were homogenously distributed onto sterilized Petri dishes. The microorganisms were individually introduced on the surface of the agar plates. Blank sterile discs, measuring 6.4 mm in diameter, were soaked in a known concentration of the test compounds and then implanted on the surface of the agar plates. A blank disc was soaked in the solvent (DMSO) and implanted as a negative control on each plate along with the standard drugs. The plates were incubated at 37 °C (24 h) and 27 °C (48 h) for bacterial and fungal strains, respectively.

## 4. Conclusions

Three Schiff base ligands containing the azo group, 1-{3-[(3-hydroxypropylimino) methyl]-4-hydroxyphenylazo}-4-nitrobenzene, 1-{3-[(3-hydroxypropylimino)methyl]-4-hydroxyphenylazo}-2-chloro-4-nitrobenzene and 1-{3-[(3-hydroxypropylimino)methyl]-4-hydroxyphenylazo}-4-chloro-3-nitrobenzene were synthesized. The ligands and their complexes with copper(II) and cobalt(II) ions were characterized by elemental analysis, FTIR spectroscopy, UV-Vis spectroscopy, ^1^H-NMR spectroscopy and thermogravimetric analysis. The room temperature effective magnetic moments of complexes are 1.45, 1.56, 1.62, 2.16, 2.26 and 2.80 B.M. for comlexes **3a**, **3b**, **3c**, **4a** **4b**, and **4c**, respectively. These values indicate that the dimetallic complexes are paramagnetic with considerable electronic communication between the two metal centers. The cobalt(II) and copper(II) complexes are coordinated by the N azomethine and O phenoxyl atoms of the ligands. In addition, the Schiff base ligands and their metal complexes were evaluated for their *in vitro* antibacterial activity using the disc diffusion method.
